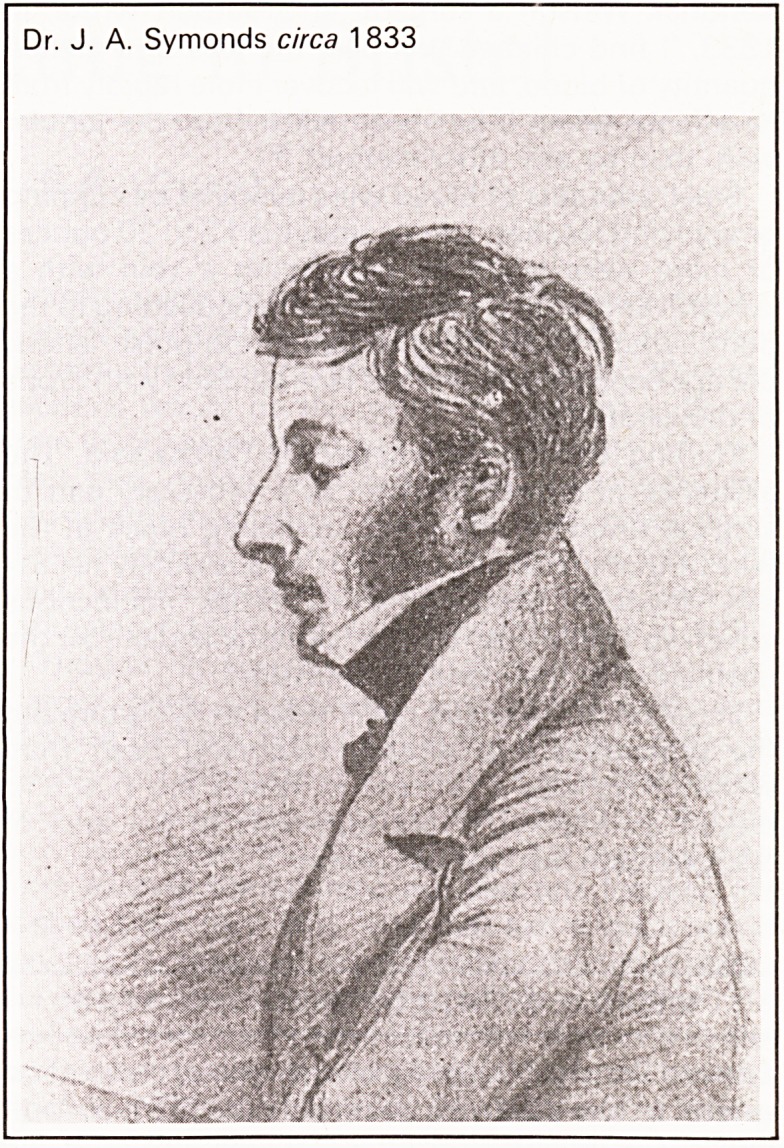# The First B.M.A. Meeting in Bristol

**Published:** 1983-07

**Authors:** Patricia Craig


### The First B.M.A. Meeting in Bristol

**Patricia Craig, M.B., B.S., M.R.C.S., L.R.C.P., B.A. (Open)**

The British Medical Association originally called the Provincial Medical and Surgical Association was founded by Charles Hastings and held its first meeting at Worcester, where he was physician to the Infirmary in July 1832. The second meeting took place in Bristol on 19th July 1833.[1]

The membership of the Association grew in its first year from around one hundred to just over three hundred. It was because so high a proportion of members, about eighty, lived in Bristol, Bath and the neighbouring country towns and villages that Bristol was chosen as venue for the Association's second meeting.[2] An added reason was that Edward Barlow, friend and collaborator of Charles Hastings, practised as a physician in Bath.[1] This short account of the meetings and some of the participants, will, it is hoped, give readers some idea of the state of medical practice in the earliest days of the B.M.A.

### FIRST ANNIVERSARY MEETING OF P.M.S.A.

On the 26th June 1833 the following entry was made in the minutes of the Weekly Committee of the Bristol Infirmary;

'Dr. Carrick and Mr. Hetling request the favour of the committee to permit the P.M.S.A. to hold their morning annual meeting in the committee room of the Infirmary on the 19th of July next.'

On the 19th July, from 11 a.m. onwards medical men began to arrive at the Infirmary which they were able to inspect; it was the object of 'general attraction and approbation'.[2]

At 1 p.m. an address was given by the President, Andrew Carrick, senior physician to Bristol Infirmary. After a short business session in which satisfactory finances and growing membership were noted Dr. Barlow of Bath gave an address. The meeting then adjourned and reconvened at 6 p.m., for dinner at Ivatts Hotel.[3]

### THE PRESIDENTIAL ADDRESS

Andrew Carrick was born in Stirling in 1767. He studied in Glasgow and at Edinburgh, where he graduated, then at the Hotel Disu, Paris and in Rome. He settled in Clifton in 1795 and was appointed to the Infirmary in 1810.[4] In his time the 'heroic method of treatment was in the ascendant' Henry Alford, Taunton surgeon and former pupil of Carrick, recalls. Carrick was grave and kindly but his treatments were violent. On his outpatient morning up to 20 venesections would be requested. In-patients suffering from fever, rheumatism and acute inflammation were bled and dosed with purgatives, mercurials and nauseating medicines.[5] Carrick was an advocate of the Hotwells Springs whose popularity as a cure he tried, unsuccessfully to revive.[6] He retired from the Infirmary in 1834 and died, a wealthy man in 1837.

Though he was an elderly man at the time Dr. Carrick gave an address[3] which was lively and forward looking. He said that the happiest times of his life had been spent in cordial social intercourse with colleagues and he expressed his delight that the new association would bring together medical men from all over the country, not just one locality. He looked back to a time 'and that not a great while beyond the scope of my remembrance' when medical men 'lived in hostile rivalry with one another'. He looked forward to a time when the divisions in the profession would be removed though as a practitioner of 59 years standing, he despaired of seeing the day. The practitioners of medicine had been divided into three orders, Physicians, Surgeons and Apothecaries but by the 1830's the new concept of the General Practitioner was beginning to be accepted. This is perhaps what Carrick meant when he alluded to a profession divided into three or even four parts. The P.M.S.A. worked to obtain a single basic standard of qualification for all doctors as well as to raise the status of provincial practitioners. Carrick was particularly aware of the distinction between Physicians and Surgeons and pointed out that the Physicians needed to know anatomy and Surgeons how to treat medical ailments. He chose a very significant example to illustrate the problems created by too strict 'job demarcation'.

'How often must every physician have had cause to regret the loss of precious time in sending for a surgeon to perform the simple but all important operation of blood letting out of delicacy to the surgical department.'

In the belief that many illnesses are caused by an excess of blood, ruthless blood letting was the order of the day. The need for it was carefully assessed on principles which were scientific and logical, saving only their false premise. In nearly every account of illness at that time, whether from the point of view of doctor or patient, there is reference to bleeding.[7]

Kendrick Watson a surgeon at Stourport wrote, in 1833, 'I find children will bear the loss of a greater quantity of blood, and will recover more rapidly from its effects, when it has been taken from the jugular vein, than by any other method'.[8]

Small amounts of blood were removed by cupping or applying leeches. Larger amounts 12 to 20 ounces or more were taken by puncturing a vein with a lancet.[9] Enthusiasts even pressed blood letting to the point 'when under the evacuation the pulse falters, the lips become pale and the face studded with drops of perspirations'.[10]

Turning to medical education Dr. Carrick said, 'The existence of apprenticeships as a necessary part of surgical tuition is the great stumbling block in the way of that uniformity which is so absolutely necessary towards breaking down those distinctions which so fatally obstruct the harmony and impair the usefulness of the medical profession'.

Legal reform was still many years in the future but the profession was already reforming itself from within. This was a process in which the P.M.S.A. played an important role especially in the area of postgraduate medical education.

Dr. Edward Barlow

### MEDICAL EVENTS OF PRECEDING YEAR

Edward Barlow (Figure 1) who reviewed the medical events of the past year, was born in 1781 at Mullingar, Co. Meath, Ireland, where his father was a Physician. He studied medicine in Dublin, Edinburgh and London and practised in Dublin until 1807 when he moved to Bath where he lived in Sydney Place, Bathwick. He had a good private practice and gave detailed attention to his patients at the United Hospital to which he was appointed in 1821 and at the City Dispensary. He founded the practical Flannel Waistcoat Charity 'to meet the want of sufficient clothing'.[11] Barlow was a member of the Medical and Chirurgical Society of London to which his friend Spurzheim, the phrenologist, also belonged.[12] In Bath, Barlow founded a society to study phrenology: it was a respectable science at the time.[13] Barlow's obituarist makes the ambiguous statement that 'he supported every measure, legal or otherwise, to advance the respect and effectiveness of the medical profession'. Dr. Barlow was secretary of the Bath branch of the P.M.S.A. from its foundation in 1836 until after it united with the Bristol branch in 1842. He wrote meticulous minutes in his beautiful handwriting. When he died in 1844, he was acclaimed by the members 'The father of our Institution. The zealous Guardian of its rights. As long as the P.M.S.A. exists the memory of Dr. Barlow will be embalmed in the affectionate remembrance of all its members'.[14]

In his retrospective address,[3] Dr. Barlow first explained, at unnecessary length, in the custom of the time, how profoundly unworthy he was of the honour bestowed upon him. He went on to praise the progressive purposes of the P.M.S.A. and of its founder, Charles Hastings. He urged the need for the application of scientific method to the study of medicine. He remarked upon the recent formation of the British Association for the Advancement of Science.[15] Chemistry had contributions to make to physic and so had meteorology. The latter was part of the subject of medical topography promoted by the P.M.S.A. The miasmatic theory of the causation of epidemics prevailed in 1833 and this made questions of climate particularly important in medical studies. Barlow made this observation, 'In a tolerably wide range of dispensary practice, I oftentimes find that for weeks together, no case calls for blood letting, when all at once an inflammatory character presents itself and numbers imperatively require the lancet on the same day'. This might, he thought, be due to electrical changes in the atmosphere. This was something he supposed might be easily elucidated whereas he believed that the recent cholera epidemic had been an act of God, which doctors should not have been expected to control, 'to arrest the course of such dispensations is as hopeless as it is irrational'.

Dr. Barlow went on to praise the 'Cyclopaedia of Practical Medicine', 'Dictionary of Practical Medicine' and 'Hospital Reports of Dr. Bright', published that year.

He pointed out the advantages of the establishment of Provincial Medical Schools, which the P.M.S.A. promoted, without alluding especially to that in Bristol, which was to open in the following autumn. Also of great significance for medical education had been the passage of the Anatomy Act in 1832. The Act made legal the provision of bodies for dissection.

Amongst those who had died since the last meeting he paid respect to his friend Spurzheim, the phrenologist, Cuvier, Bentham and Sir Everard Home.

Finally he discussed the need for medical reform. He was particularly critical of the role of the London Royal Colleges, as was the P.M.S.A. as a whole. He finished by commenting on the Association's aims, 'Kindly and friendly feelings must be promoted; talents called forth, zeal excited; science advanced; and in consequence, the public good proportionately advantaged'.

Dr. Barlow had started his address by urging the need for doctors to use and value scientific method, in the modern sense. He went on to show that in the diagnosis and treatment of disease he adhered to an 18th century system which owed more to Greek philosophy than to inductive science. Finally, in discussing cholera he made it plain that as a devout Christian and man of his time he accepted the absolute power of God and believed the bible to be literally true. Dr. Barlow was not alone in being unaware of any conflict between these points of view; this was usual in the early 19th century though it was to become more and more difficult in the next few years to reconcile old and new world views.

### BRISTOL IN 1833

In the 'Transactions of the P.M.S.A.' Vol. 2 in which the proceedings of the 1st Anniversary meeting were published, there are four other papers by Bristol men, 'Medical Topography of Bristol' by Andrew Carrick and John Addington Symonds (Figure 2),[16] 'Observations on the treatment of syphilis without mercury' by Thomas Green,[17] 'On dislocation of the shoulder' by W. F. Morgan,[18] and the paper by Henry Riley mentioned in a recent number of this journal 'A description of the anatomical structure of the liver of a rat, from Cuba'.[19] Of these the first is of particular interest because it paints a vivid picture of Bristol in 1833.

Dr. J. A. Symonds *circa* 1833

Carrick and Symonds divide their account[3] of Bristol into four parts, the physical geography, the man-made environment, the people and their occupations, and finally the diseases which prevail.

They give a very clear account of the physical situation of Bristol and explain how this accounts for its favourable climate.

Their description of the housing of the poor in Bristol is based upon the first hand experience of Symonds who had made a survey of the City prior to the cholera epidemic and had been secretary to the board directing medical care during the outbreak of the disease in 1832.

'...we find courts and close alleys very frequent. As if the original object had been to make every inch of ground available, houses may be observed in some of these courts, with their faces opposed to each other, at a distance of five or six feet only, the entrance to the area being under an archway from some street only a little less confined than the court itself. On looking at them and considering the filthy, careless habits of the occupants, the medical observer is puzzled to imagine how any degree of health can be preserved in places where exhalations from the soil and every description of human miasmata must be almost constantly detained and concentrated.'

These courts were entirely enclosed on three sides and apparently scarcely open to the sky. In the yard, animal and vegetable refuse was heaped up. Conditions in the low lying old town had been made even worse by the recent construction of the floating harbour. This had restricted the outflow from the River Frome, '... loaded with the contributions which it has received at every step of its progress through some of the most closely built and densely crowded districts. Unhappily the current of this river is narrow, torpid and scanty, in consequence of which it often struggles ineffectually with the burthens accumulated upon it and deposits them upon its bed, the sides of which become elevated into pillows for the exhausted and almost stagnant waters, and exhale miasms sufficient, it might be imagined, to infect the whole neighbourhood ... it is almost impossible to cross the bridges by which it is concealed from sight in the midst of streets and lanes, without being reminded, by particular odours, of its propinquity.'

The diet of the poor was meagre, and it was particularly regretted that they were prejudiced against oatmeal. Sometimes the food was worse than inadequate, '... we have had the pain to see meat hanging in the shops, black in colour and almost liquid in consistency'. The sweepings of greengrocers shops were eaten, inadequately boiled. In addition, spirit drinking was prevalent, partly perhaps for warmth, for clothing was insufficient.

The Irish were the poorest of the poor. Some lived in houses where each floor was let, then each room sub-let, then each corner of each room rented out to a tenant! 'Thirty individuals had, on one night, slept in a room not exceeding 20 ft. by 16 ft.' The cholera 'swooped down on nine out of the thirty and seven became corpses in a few hours'.

This was Bristol in 1832, not much superior it seems to the Manchester which Engels described about the same time.[20]

The description of the diseases prevalent in the City is not easy to follow, even when described by these excellent writers, for we find their concept of illness very difficult to understand. They had no idea of the specificity of disease nor of the germ theory. The almost visible and palpable miasms in low lying parts of Bristol must have seemed an obvious cause of illness. 'Disorders originating in malaria' were not seen nearer than Bridgewater. Post mortems showed most fever victims had intestinal ulceration, so typhoid was probably more common than typhus. Bronchitis, pleurisy, phthisis and scrofula were common and so was rheumatism in both acute and chronic form. 'Gastric derangements which pass under the denomination of pyrosis, gastralgia, morbid sensibility, etc.' were often seen as were 'females labouring under some form of hysteria, considering this term generic for all those neurotic, atonic, anomolous ailments, to which females are so obnoxious'.

Some calculations showed Bristol to be healthy by comparison with a number of cities. There was a very high infant death rate but this was usual at the time.

### HOW SOME B.M.A. MEMBERS APPEARED TO AN OUTSIDER

The papers published in the Transactions and the account of the proceedings at their First Anniversary meeting tell us little about the day to day work of the P.M.S.A. members, and nothing of how others saw them. The diary of the Rev. John Skinner[20] of Camerton does something to fill this gap for his family and his parishioners were patients of several of the gentlemen assembled at the Bristol Infirmary on 19th July 1833. The 'Dr Garrick' who attended Skinner's wife and daughter, Laura, at Clifton was certainly Andrew Carrick, the P.M.S.A. President. George Norman, the surgeon from Bath, who was in Bristol on 19th July 1833, attended Skinner's mother and brother in Bath. When Joseph, Skinner's son, developed phthisis he did not, like his mother and sister, consult a Bristol physician, but went instead to Norman of Bath who readily treated his 'medical' complaint. There was no difference in the treatment. 'Poor Joseph was blooded on Saturday' wrote Skinner in July 1832. Laura had been bled and forbidden to eat meat; neither child recovered. Skinner continued to take medical advice but not uncritically.

'What a system do gentlemen of the lancet now pursue in cases of inflammation! There appears to be little chance if the disorder be violent and can alone be remedied by copious draughts of the vital stream. The only difference seems to be the patient may die quiet instead of quitting the world in a raging fever.'

The sufferings of poor Garratt, the miner, whose back was broken by a fall of coal were made worse by disagreements between Curtis, the doctor of the club, and Mr. Flower, the surgeon from Chilcompton.

'Mr. Crang, I find is no less at enimity with Mr. Flower than Curtis. These doctors differ among themselves but it is hard that their patients should suffer for their disputes.'

Mr. Crang was the apothecary from Timsbury who was consulted by the Skinner family at home and who treated many of the parishioners. He had been in practice before 1815 and so was able to register in 1859, even though he had no paper qualification. Mr. Flower had obtained the M.R.C.S. in 1808. Both men were at the Bristol meeting and both became very active members of the Bath and Bristol branch of the P.M.S.A. and served as its president.[14] Skinner was sometimes exasperated by doctors but he regarded them, unlike Methodist lay preachers, as fellow professionals. When a young man told Skinner he would as soon listen to a miner with a gift from God, as to an Anglican parson, Skinner asked him 'if he had injured any part of his body or had any inward complaint, would he send for a regular bred surgeon to attend him such as Mr. Crang or Mr. Flower or go to old Crow the horse doctor at Radstock'.

John Skinner died in 1839, too soon to see great change in medical practice. Henry Alford, the pupil of Carrick, who was at the meeting on 19th July 1833, wrote in 1890.

'So great is the change in the theory and practice, both of medicine and surgery, since the date of which I am writing (1822–28) that my record has almost an archaeological interest.'[5]

Between 1800 and 1900 the practice of medicine underwent a fundamental change of structure, scientific, legal and social. The B.M.A. had as its original purpose the promotion of medical reform. The founders saw as the most important means of attaining this end the increasing professional competance of members. The most important function of the Association was to provide, at regular national and later also local meetings, a forum for post-graduate education. The Association continued to be well supported in the Bristol district;[22] the 1833 meeting was the first of many in the city.

### REFERENCES

1. LITTLE, E. M. (1932) History of the British Medical Association. 1932. *B.M.A.*
2. Felix Farley's *Bristol Journal* quoted by the *Lancet* **24**, 1832–1833.
3. The proceedings of the meeting and Dr. Barlow's address are published in *The Transactions of the Provincial Medical and Surgical Association (T.P.M.S.A.)* Vol. 2, 1833. Dr. Carrick's address had been circulated separately to Members. It was published in local papers and reprinted in *Lancet*, Vol. 24.
4. MONRO SMITH, G. (1917). History of the Bristol Royal Infirmary.
5. ALFORD, H. (1890) The B.R.I. in my student days. *Bris.Med.Chir.J.* September.
6. LATIMER. Entry for 1816. Annals of Bristol in Nineteenth Century.
7. See, for example, works by Edward Barlow to whom reference is made below. 'On the objects and modes of medical treatment'. *T.P.M.S.A.* Vol. 1. 'Records of ovarian tumours'. *T.P.M.S.A.* Vol. 4—where he gives details of schedules of treatment. 'An essay on the medicinal efficacy and employment of the Bath waters'. Bath, 1822—in which he records deriving benefit from being bled himself, of up to 40 ounces at a time.
8. WATSON, K. (1833) A topographical account of Stourport. *T.P.M.S.A.* Vol. 2.
9. ROLLS, Dr. R. (1983) Personal communication.
10. BARLOW, Dr. E. (1822) Essays on Bath Waters.
11. *Bath Herald*, 6th April.
12. *Trans.Med.Chir.Soc. of London* 1832.
13. CLARKE, E. and DEWHURST, K. (1978) An illustrated history of brain function.
14. Minutes of the Bath and Bristol Branch of the P.M.S.A. 1844. The Bath branch was formed in 1836 and the Bristol branch in 1839. They amalgamated in 1842.
15. British Association founded in 1831.
16. Andrew Carrick see above. John Addington Symonds, 1801–71. Physician to the Bristol General Hospital from its foundation in 1831 until 1845. After his father's death, Symonds' son edited a collection of his father's essays 'Miscellany', to which he contributed a biographical foreword.
17. MONRO SMITH, G. History of the Bristol Royal Infirmary. (1917) Thomas Green 1801–78. Surgeon B.R.I. 1844–64. Bristol: Arrowsmith.
18. MONRO SMITH, G. (1917) History of the Bristol Royal Infirmary. William Francis Morgan 1800–72. Surgeon B.R.I. 1837–54.
19. *Bristol Med.Chir.J.* July–October 1982.
20. ENGELS, F. Condition of the Working Class in England in 1844.
21. SKINNER, J. (ed.) COMBS, H. and P. (1971) Journal of a Somerset Rector 1803–34. Bath: Kingsmead Press. There was no 'Dr. Garrick' in Clifton in Skinner's time. A 'C' could easily be mistaken for a 'G'. Most conclusively G. Monro Smith states that Carrick's wife was a 'Miss Tudway' and Skinner tells us (p. 132) that Dr. Garrick's wife was formerly a Miss Tudway of Wells. George Norman was a well known surgeon in Bath, M.R.C.S. 1801. He, like Morgan and Green of the B.R.I., was amongst the first 300 fellows of the R.C.S. of London when the Fellowship was instituted in 1843.
22. Members included most of Bristol's best known doctors. Richard Smith was a Vice-President at the 1833 meeting. William Budd was president of the local branch in 1856, the year in which the name was changed from P.M.S.A. to B.M.A.

## Figures and Tables

**Figure f1:**
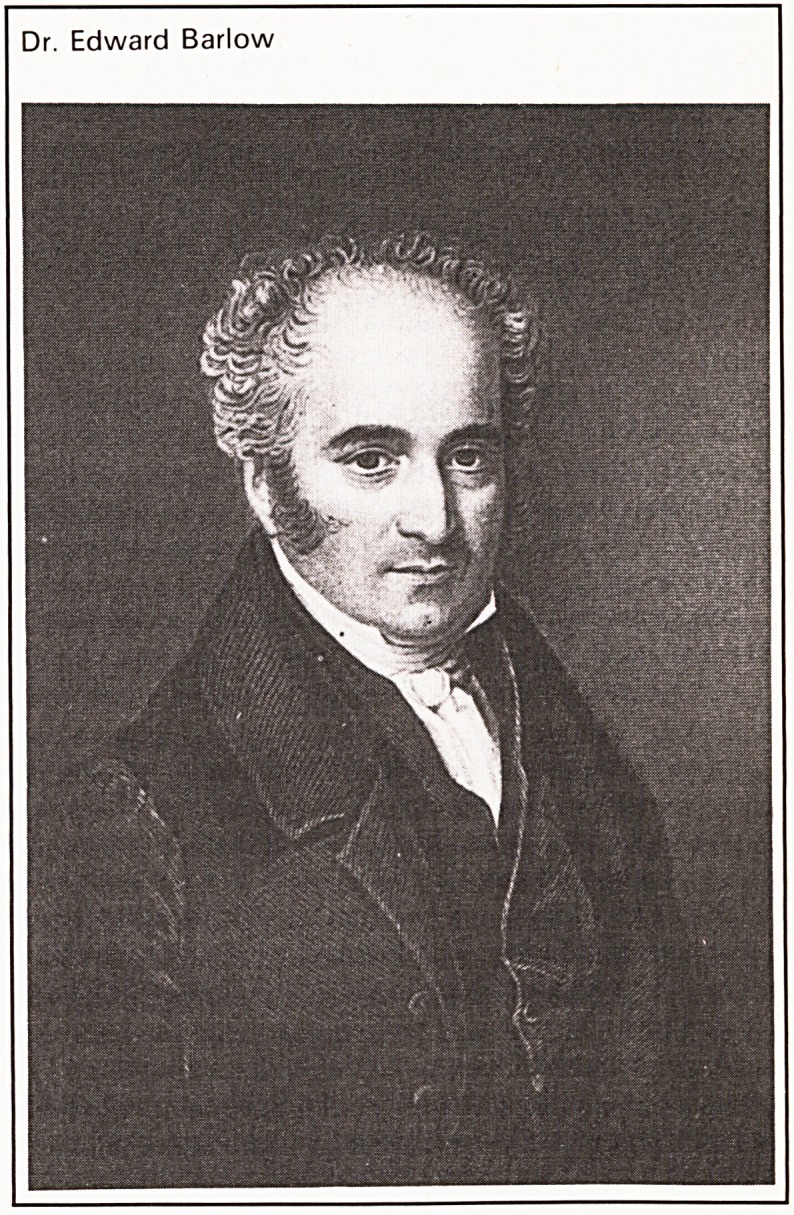


**Figure f2:**